# The Regulation of *Staphylococcus aureus*-Induced Inflammatory Responses in Bovine Mammary Epithelial Cells

**DOI:** 10.3389/fvets.2021.683886

**Published:** 2021-05-31

**Authors:** Mingcheng Cai, Wenqiao Fan, Xiaoying Li, Hanchang Sun, Liuliu Dai, Defang Lei, Ying Dai, Yuhua Liao

**Affiliations:** College of Landscape Architecture and Life Science/Institute of Special Plants, Chongqing University of Arts and Sciences, Yongchuan, China

**Keywords:** mastitis, LTA, mammary epithelial cells, miR-23a, exosome

## Abstract

Mastitis, an inflammatory disease, causes severe economic loss in the dairy industry, which is mainly infected by bacteria. *Staphylococcus aureus* (*S. aureus*), the major pathogenic microorganism, derived from lipoteichoic acid (LTA) has been identified to activate inflammatory responses, but the cellular or intercellular regulatory mechanism is unclear. This study mainly focused on the effects of LTA in bovine mammary epithelial cells (Mac-T) and elaborated the regulation of microRNAs (miRNAs). The results showed that LTA enhanced the messenger RNA (mRNA) expression and production of tumor necrosis factor α (TNF-α) and interleukin (IL)-6. Furthermore, LTA could activate Toll-like receptor (TLR)2/MyD88-mediated phosphoinositide 3-kinase (PI3K)/AKT pathway, and TLR2 plays a pivotal role in LTA-induced inflammatory responses. The results of qRT-PCR showed that miRNA levels increased and reached the highest at 3 h and then gradually decreased over time in Mac-T cells. In exosomes, the levels of 11 and three miRNAs were upregulated and downregulated at 24 h, respectively. In addition, miR-23a showed the highest increase in Mac-T cells treated with LTA and targeted PI3K to regulate inflammatory responses. Furthermore, Mac-T cell-derived exosomes were identified to play a cell–cell communication by promoting M1 polarization of bovine macrophages. In summary, our study demonstrated that LTA could activate inflammatory responses via TLR2/MyD88/PI3K/AKT signaling pathway, and miR-23a inhibited it by targeting PI3K. Furthermore, we found that Mac-T cell-derived exosomes might be associated with inflammatory responses by promoting M1 polarization of bovine macrophages.

## Introduction

Mastitis is one of the most common diseases in the dairy industry, which causes ~2-billion-dollar losses a year due to reduced milk production and changes in milk composition, treatment, veterinary costs, and replacement animal costs in the US (1). As a group, *Staphylococcus aureus* are the most prevalent (ranging from 9.1 to 16.6% of milk samples) causative agents of bovine mastitis in many regions and typically more isolated from subclinical than from clinical cases of mastitis (2–4). However, the long-term usage of antibiotics leads to multidrug resistance in bacteria, including *S. aureus*. Accordingly, 70~90% of *Staphylococcal* mastitis in cattle are resistant to antibiotic treatment, which forces 25% of cows to stop calf-sucking (5, 6). Therefore, further analysis of the regulatory mechanism of *S. aureus*-infected mastitis is very important for effective therapies.

*S. aureus* that directly colonizes breast tissue through internalizing epithelial and endothelial cells severely affects udder structure and milk quality (7). Lipoteichoic acid (LTA), the main virulence factor of *S. aureus*, is composed of repeating polymer and glycolipid and could be recognized by Toll-like receptor 2 (TLR2) (8). Subsequently, the downstream signaling of PI3K-AKT, nuclear factor (NF)-κB, or mitogen-activated protein kinase (MAPK) pathways was activated and could induce the expression of inflammatory genes (9). In addition, some genes, such as tumor necrosis factor α (TNF-α) and basic fibroblast growth factor (bFGF), are identified to be associated with inflammation in bovine mammary epithelial cells (bMECs; Mac-T) (10). In bovine mammary gland, bMECs play an important role in bacterial infection and secrete a plethora of cytokines and other inflammatory mediators after being stimulated by bacterial toxins, such as cell wall components, to cause mastitis. Therefore, it is particularly important to reveal the mechanisms of host immunity in bacteria-infected mammary epithelial cells. Additionally, few studies focused on the regulatory mechanisms of TLR2-mediated activation of PI3K-AKT pathway in LTA-infected bMECs.

In the last decades, many studies focused on the regulation of microRNAs (miRNAs) in inflammation and found some key and well-studied miRNAs, such as miR-21, miR-155, and miR-146a, which regulate the activation of NF-κB in inflammatory responses (11). However, the regulatory mechanisms of miRNAs in bacteria-induced immune and inflammatory responses in dairy cattle are still not well-understood. Many studies identified a large number of differentially expressed miRNAs in infected vs. healthy mammary tissue or bMECs, such as miR-223, miR-15a-3p, miR-146a, miR-146b, and miR-23a (12–14). Although part of miRNAs is identified in dairy cows, the current understanding in the molecular levels between miRNAs and mastitis is far from comprehensive. It is known that miR-23a is highly conserved across species and can regulate several disease processes, such as immune regulation, inflammation, and cancer (15, 16). It is identified that miR-23a modulates inflammatory responses via directly targeting TXNIP/NLRP3 inflammasome axis (17). However, the regulatory mechanism of miR-23a in bovine mastitis is still unknown.

In addition, miRNAs have been identified in fluids, including cell supernatant, blood, and milk, and many studies have identified that miRNAs, in biological fluids, are encapsulated in different vesicles, such as exosomes (18, 19). Exosomes are endogenous nanovesicles (30~150 nm) that carry various cargoes, including proteins, nucleic acids, and lipids, to specific targeted cells, and the released contents may trigger the intracellular signals. Previous studies showed that various epithelial cells, such as mammary epithelial cells, could participate in the regulation of immunity through secreting exosomes (20). It was suggested that the cytokines or other inflammatory mediators released by mammary epithelial cells could be transferred to the neighboring cells, such as macrophages, which might be activated (7). Additionally, mammary epithelial cells could interact with macrophages in another way. For example, breast cancer-derived exosomal glycoprotein 130 induces a pro-survival phenotype in macrophages by activating the interleukin (IL)-6 response pathway (21). In epithelial ovarian cancer, epithelial cell-derived exosomes transport miRNAs, such as miR-21-3p, miR-125b-5p, and miR-181d-5p, to macrophages, which polarized toward M2 (22). Therefore, it is possible that the miRNAs circulating in exosomes can be studied as intracellular regulation during bovine mastitis.

Our previous study has identified 14 significantly differential miRNAs in *S. aureus*-infected bovine milk-derived exosomes, which are involved in inflammation and immunity (23). In this study, we identified the miRNA levels in bMECs and cell-derived exosomes treated with *S. aureus*-derived LTA. And we focused on the regulatory mechanism of cellular miRNAs and the communication between bMECs and macrophages via exosomes.

## Materials and Methods

### Cell Culture

Mac-T cells were recovered and cultured in Dulbecco's modified Eagle's medium (DMEM; HyClone) medium supplemented with 10% fetal bovine serum (FBS, Gibco) and 1% antibiotics (Invitrogen) at 37°C with 5% CO_2_. Bovine peripheral blood macrophages were isolated from bovine blood as described previously (24). Briefly, cells were isolated by centrifugation (2,500 × *g* for 20 min), and purified with Ficoll gradients (Solarbio, China). Then, the layer with macrophages was removed and cells were cultured in RPMI 1640 (10% FBS), supplemented with 50 mM 2-mercaptoethanol, 5% non-essential amino acids, 100 mM sodium pyruvate, and 1 M HEPES solution. Then, cells were incubated at 37°C in the presence of 5% CO_2_ with 100 ng/ml granulocyte–macrophage colony-stimulating factor (GM-CSF; abcam, USA) for 3 days, then the medium was replaced with fresh culture medium with GM-CSF for 5 extra days to induce cell differentiation into macrophages.

### Lipoteichoic Acid Treatment

Mac-T cells were cultured to a density of 80%, then the medium was replaced with DMEM with 10% exosome-free FBS. Subsequently, LTA (Sigma-Aldrich, USA) was added into the medium with a concentration of 40 μg/ml to construct the inflammatory cell model. After stimulation for 0, 1, 3, 6, 12, and 24 h, the supernatants were discarded and replaced with DMEM with exosome-free FBS at different time points, respectively. The cells were further incubated for 24 h, and the culture supernatants and cells were collected for other studies.

### Inhibition of Toll-Like Receptor 2

For conducting the analysis, cells were placed into six-well plates and cultivated to monolayer formation. Then, 30 μg/ml of 1-palmitoyl-2-arachidonyl-*sn*-glycero-3-phosphorylcholine (OxPAPC; InvivoGen, USA) was added to cells for 1 h, following with LTA treatment.

### Enzyme-Linked Immunosorbent Assay

At different time points, 0, 1, 3, 6, 12, and 24 h, the supernatants in LTA-treated Mac-T cells were collected, followed by centrifugation (1,500 × *g*, 4°C, 15 min) to remove cell debris. The concentration of TNF-α and IL-6 secreted in supernatants was measured with appropriate ELISA kits (Cusabio, China) according to the manufacturer's instructions.

### Exosome Isolation and Particle Size Identification

Before exosome collection, the Mac-T cells were cultured in DMEM containing exosome-free FBS, which was centrifuged at 100,000 × *g* to remove exosomes. Then, the supernatants were collected with 50-ml centrifuge tube for further sequential centrifugations. Briefly, the supernatants were firstly centrifuged at 800 × *g* for 10 min to pellet the residual cells, and the supernatants were further centrifuged at 12,000 × *g* for 20 min to remove cellular debris. Finally, cell-derived exosomes were pelleted from the supernatant with centrifugation at 100,000 × *g* for 2 h. The exosomes were resuspended with PBS to remove the impurity twice and resuspended with PBS for further studies.

### Transmission Electron Microscopy

Transmission electron microscopy of membrane exosomes was performed using a standard technique as previously described. Briefly, about 10 μl prepared exosomes were placed on a slide, then the carbon-coated copper grid was lightly covered by the droplet and kept for 10~30 s. Subsequently, the grid was then removed, and the remaining liquid was sucked out with a piece of sterile filter paper. The same side of the grid was covered by a drop of 2% phosphotungstic acid, pH 7.0, for 5 s, and the excess liquid was removed. After several minutes, the grid was dry and then examined with Tecnai G2F20 S-TWIN (FEI, Hillsboro, USA) (25).

### Transfection of miRNA Mimic and Inhibitor

Mac-T cells were seeded in six- or 24-well plates until reaching 80% confluence and transfected with synthetic mimics (50 nM) and inhibitors (100 nM) of miR-23a (Sangon, Shanghai, China) by using Lipofectamine 3000 (Invitrogen, Carlsbad, CA, USA) according to the manufacturer's instructions, respectively. miRNA mimic control (NC) or inhibitor control (INC) were transfected and used as negative controls. After culturing for 6 h, cells were then treated with LTA.

### Flow Cytometric Analysis

Macrophages were treated with the indicated reagents for 24 h and stained in PBS containing 0.5% bovine serum albumin (BSA) with specific antibody, CD11b (BD Pharmingen, USA), for 15~30 min at 4°C in the dark. After washing, the cells were analyzed by fluorescence-activated cell sorting (FACS) (BD Biosciences, USA).

### RNA Isolation and Real-Time Quantitative Polymerase Chain Reaction

Total RNA was extracted from all samples, Mac-T and macrophages, by using TRIzol Reagent (Invitrogen, USA). NanoDrop 2000 UV-Vis Spectrophotometer and agarose gel electrophoresis were used to identify the purity and integrity of RNA. Exosomal RNA was isolated by using the miRNeasy Mini Kit (QIAGEN, Germany). cDNA was amplified with PrimeScript RT reagent Kit and Mir-X^TM^ miRNA First-Strand Synthesis Kit (TAKARA, Japan). Real-time quantitative polymerase chain reaction (RT-qPCR) was performed with SYBR Premix Ex TaqII (TAKARA, Japan) on CFX96 Real-time system (BioRad, USA). Relative fold changes of messenger RNA (mRNA) and miRNA were calculated using the 2^ΔΔCt(ΔCtsample−ΔCtcontrol)^ method, and glyceraldehyde 3-phosphate dehydrogenase (GAPDH) and U6 were used as housekeeping genes, respectively. The RT-qPCR was performed in a 20-μl reaction system, and the information of primers is shown in Table 1.

**Table 1:** Primer sequences used for qRT-PCR.

**Gene**	**Primer sequence (5^**′**^-3^**′**^)**	**Product Size (bp)**
TLR2	F: AAGCTGCGGAAGATCATGAACACC R: TAGAAGGACCACCACCAGACCAAG	150
Myd88	F: GCAGCATAACTCGGATAAA R: CAGACACGCACAACTTCA	177 (26)
TNF-α	F: ACCCAGCCAACAGAAGC R: CCAGACGGGAGACAGGA	175 (26)
IL-6	F: GCTCTCATTAAGCGCATGGT R: AGCAAATCGCCTGATTGAAC	172 (27)
iNOS	F: GGACTTGGCTACGGAACTGG R: GGTGAAGCGTGTCTTGGAAA	257 (27)
ARG-1	F: GGCTGATGTGGTGGCAGAAGTC R: GTGGAGTGTTGATGTCCGTGTGAG	158
GAPDH	F: TCAACGGGAAGCTCACTGG R: CCCCAGCATCGAAGGTAGA	237
U6	F: GCTTCGGCAGCACATATACTAAAAT R: CGCTTCACGAATTTGCGTGTCAT	89 (23)

*GAPDH, glyceraldehyde 3-phosphate dehydrogenase; IL-6, interleukin 6; iNOS, inducible nitric oxide synthase; TLR2, Toll-like receptor 2; TNF-α, tumor necrosis factor α*.

### Western Blot

All samples were lysed by using radioimmunoprecipitation assay (RIPA) buffer containing 1% protease inhibitors (Beyotime, Shanghai, China) for 10 min on a shaker and then centrifuged at 10,000 × *g* for 5 min at 4°C, and the supernatant was collected and stored at −80°C. The protein content was quantified with bicinchoninic acid (BCA) protein assay kit (Beyotime, Shanghai, China). Every protein sample (10 μg) was denatured at 100°C for 5 min and was resolved with 12% acrylamide gel. Then, proteins were electronically transferred onto a polyvinylidene fluoride (PVDF) membrane, and the membranes were rinsed with Tris-buffered saline with Tween 20 (TBST) and blocked with skimmed milk at room temperature for 2 h. Subsequently, the membranes were incubated with diluted primary antibodies overnight at 4°C, such as anti-CD81 (Santa Cruz Biotechnology, USA), anti-calnexin (abcam, USA), anti-Tsg-101 (Santa Cruz Biotechnology, USA), anti-MyD88 (Novus, USA), anti-PI3K (Absin, China), anti-AKT (Cell Signaling, USA), anti-p-AKT (Ser473) (Cell Signaling, USA), anti-TLR2 (Absin, China), or anti-β-actin (Proteintech, China), respectively. Then, membranes were washed 5 × 10 min with TBST and incubated with diluted secondary antibodies (Proteintech, China) at room temperature for 2 h. Finally, the membranes were washed and incubated with enhanced chemiluminescence (ECL) kit (Thermo Fisher, USA) following with the manufacturer's recommendations.

### Target Gene Prediction and Luciferase Assays

The online databases miRbase (http://www.mirbase.org/), miRwalk (http://mirwalk.umm.uni-heidelberg.de/), Targetscan (http://www.targetscan.org/mamm_31/) were used to predict target genes and hybridization between miRNA and the 3′-UTR of genes. The predicted target genes were used for Kyoto Encyclopedia of Genes and Genomes (KEGG) pathway analysis by using DAVID 6.8. The method of luciferase reporter assay is based on a previous method (28). 293T cells, seeded in 24-well plates in triplicate, were used. After the cell density reached 80%, the psiCHECK-2 plasmid (Promega) of wild-type or mutant 3′-UTR was co-transfected with synthetic miR-23a mimic or control into 293T cells by using Lipofectamine 3000 (Invitrogen, USA). After 48 h, firefly luciferase activity was measured and normalized to Renilla luciferase activity with Double-Luciferase reporter assay kit according to the manufacturer's protocol (Transgen, Beijing, China).

### Statistical Analysis

Statistical tests were performed with GraphPad Prism (v7.0) software (GraphPad Software, Inc.), and the results were presented as means ± SEM. The data of OxPAPC inhibition (**Figure 3A**), miR-23a mimic and inhibitor transfection, and exosome-treated macrophages were compared by a paired *t*-test. The data of qRT-PCR and Western blot analysis of LTA-treated cells at different time points were evaluated with Shapiro–Wilk test. After confirming that the data follow a normal distribution, a one-way analysis of variance (ANOVA) analysis was applied followed by *post-hoc* Tukey analysis. A significance level of *p* < 0.05 was considered significant.

## Results

### Lipoteichoic Acid Induced an Inflammatory Response in Mac-T Cells

**Figure 1:**
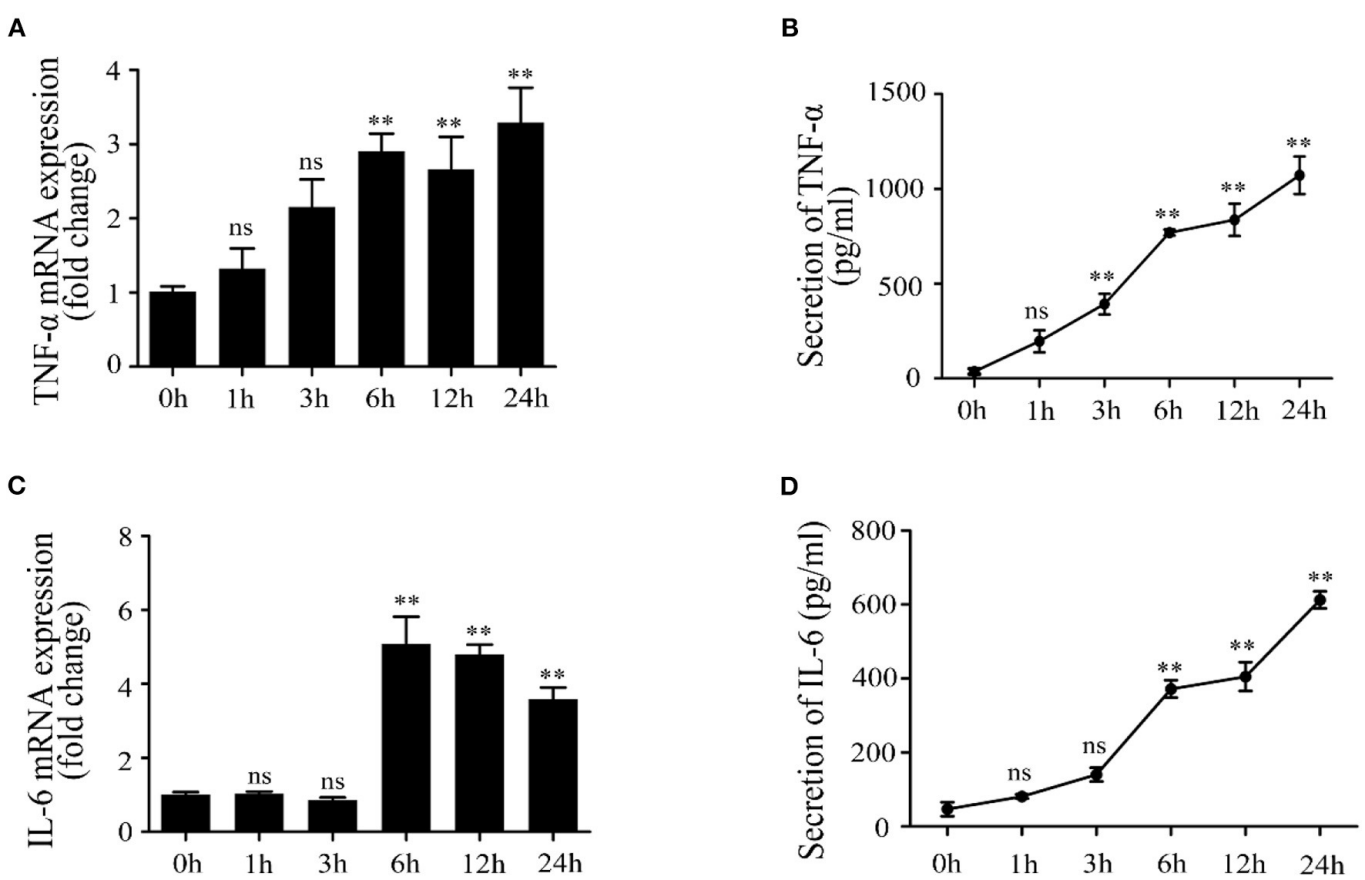
Effects of lipoteichoic acid (LTA) on the expression and production of pro-inflammatory cytokines in Mac-T cells at different time points (compared with 0 h, *n* = 3). **(A)** The mRNA expression of tumor necrosis factor (TNF)-α. **(B)** The secretion of TNF-α (pg/ml) in cell culture medium. **(C)** The mRNA expression of interleukin (IL)-6. **(D)** The secretion of IL-6 (pg/ml) in cell culture medium. **p < 0.01.

Firstly, Mac-T cells were treated with LTA for different times. The results indicated that LTA induced an upregulation of TNF-α mRNA level at 1 and 3 h but showed no significant difference (*p* > 0.05) (Figure 1A). TNF-α mRNA level significantly upregulated at 6 and 12 h (*p* < 0.05) and reaching the peak at 24 h (*p* < 0.05). The secretion of TNF-α (pg/ml) showed significant increases at 3, 6, 12, and 24 h (*p* < 0.05) (Figure 1B). In addition, the mRNA expression and concentration of IL-6 showed no significant differences at 1 and 3 h (*p* > 0.05). IL-6 mRNA level significantly increased at 6, 12, and 24 h and reached the peak at 6 h (*p* < 0.05) (Figure 1C). The secretion of IL-6 (pg/ml) increased with time and showed significant differences at 6, 12, and 24 h (*p* < 0.05) (Figure 1D).

### Lipoteichoic Acid Activates TLR2 and Induces an Inflammatory Response by TLR2/MyD88/PI3K/AKT Pathway

**Figure 2:**
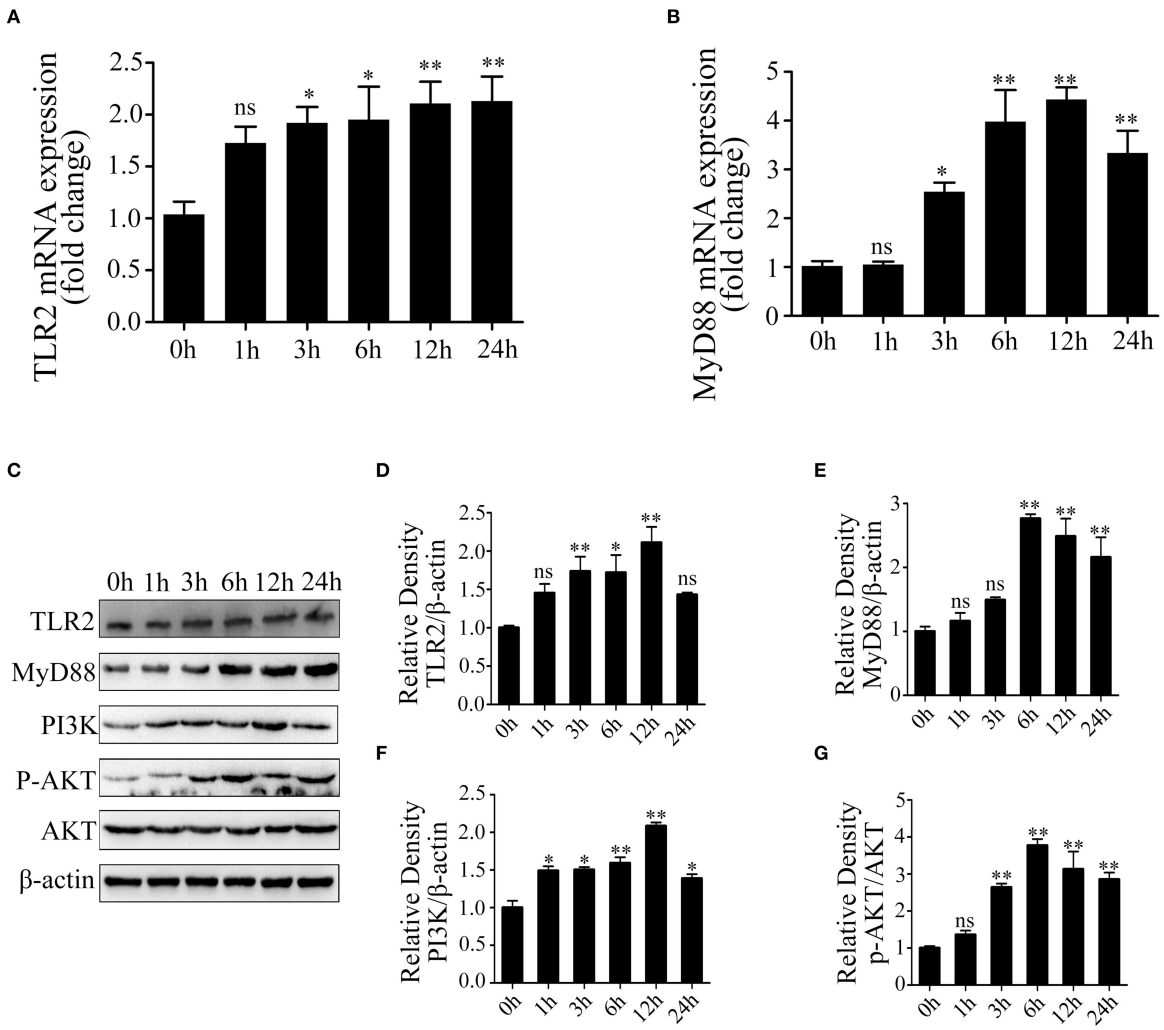
The activation of Toll-like receptor (TLR)2/MyD88-mediated phosphoinositide 3-kinase (PI3K)/AKT signaling pathway in lipoteichoic acid (LTA)-treated Mac-T cells at different time points (compared with 0 h, *n* = 3). **(A)** The mRNA expression of TLR2. **(B)** The mRNA expression of MyD88. **(C)** Western blot analysis of TLR2, MyD88, PI3K, and AKT. **(D)** The expression of TLR2 protein. **(E)** The expression of MyD88 protein. **(F)** The expression of PI3K protein. **(G)** The expression of p-AKT protein. *p < 0.05, **p < 0.01.

We next investigated the activated inflammation-related pathway in LTA-treated Mac-T cells. The result showed that TLR2 mRNA expression was significantly upregulated at 3, 6, 12, and 24 h (*p* < 0.05) (Figure 2A). Our results showed that LTA caused significant upregulation of MyD88 mRNA at 3, 6, and 12 h and then decreased at 24 h (*p* < 0.05) (Figure 2B). The expression of key genes was investigated and shown in Figure 2C. The level of TLR2 and MyD88 protein peaked at 12 and 6 h, respectively (*p* < 0.05), followed by a gradual decrease with a longer stimulation time of 24 h (*p* < 0.05) (Figures 2D,E). Furthermore, the expression of PI3K protein showed a notable increase at 1, 3, 6, and 12 h (*p* < 0.05) and peaked at 12 h in LTA-induced cells (*p* < 0.05) (Figure 2F). p-AKT protein level increased at 3 h (*p* < 0.05) and peaked at 6 h (*p* < 0.05), and then decreased at 12 and 24 h (*p* < 0.05) (Figure 2G). These results showed that LTA could activate an inflammatory response via TLR2/MyD88-mediated PI3K/AKT signaling pathway in Mac-T cells.

### Toll-Like Receptor 2 Is Necessary in Lipoteichoic Acid-Induced Inflammatory Responses in Mac-T Cells

**Figure 3:**
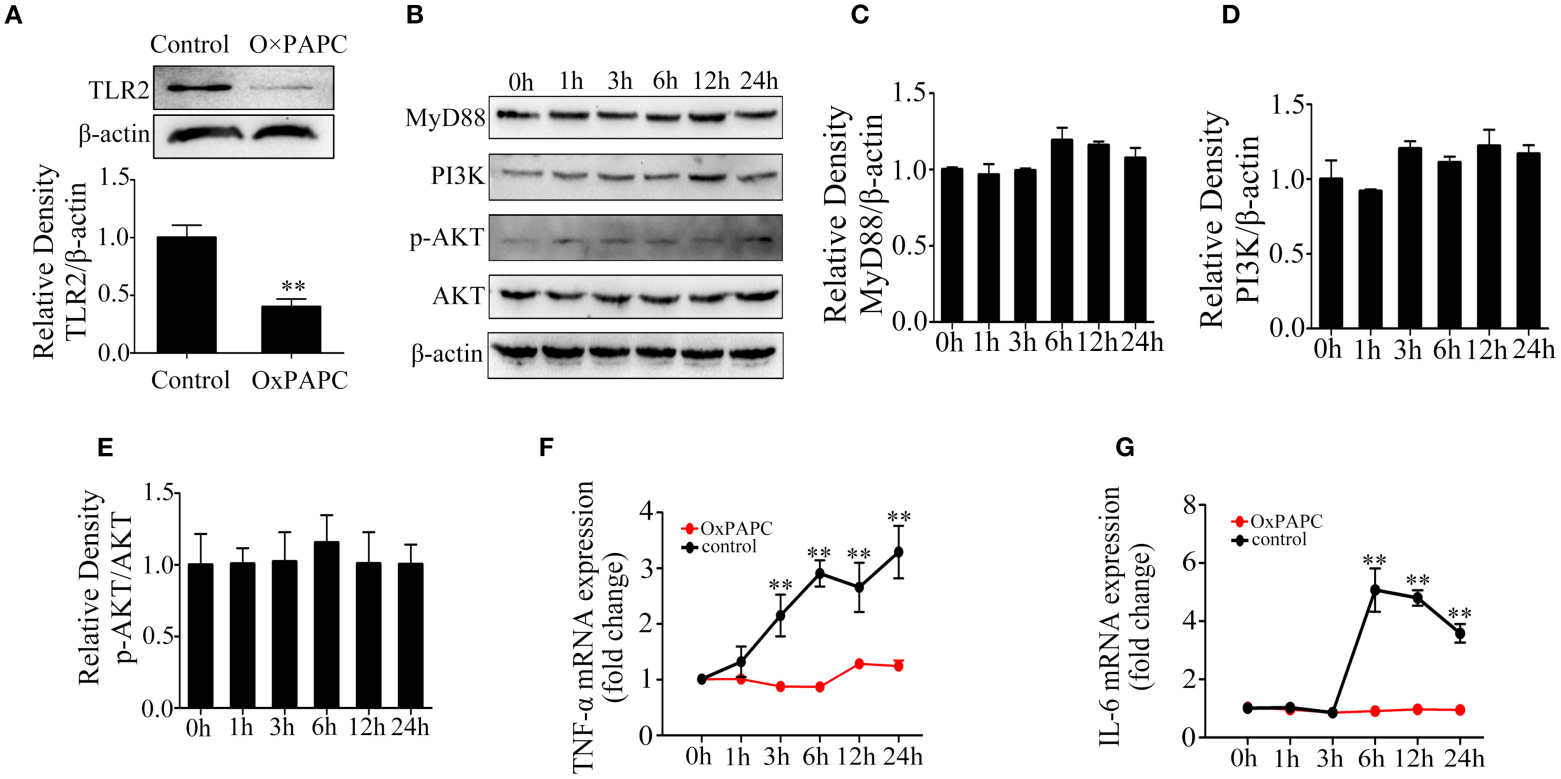
Effects of 1-palmitoyl-2-arachidonyl-*sn*-glycero-3-phosphorylcholine (OxPAPC) on lipoteichoic acid (LTA)-induced inflammatory response (compared with 0 h, *n* = 3). **(A)** The expression of Toll-like receptor (TLR)2 protein. **(B)** Western blot analysis of phosphoinositide 3-kinase (PI3K)/AKT signaling pathway. **(C)** The expression of MyD88 protein. **(D)** The expression of PI3K protein. **(E)** The expression of p-AKT protein. **(F)** The mRNA expression of tumor necrosis factor (TNF)-α. **(G)** The mRNA expression of interleukin (IL)-6. **p < 0.01.

Furthermore, to confirm the role of TLR2 in the activation of inflammatory response by LTA, Mac-T cells were treated with OxPAPC. The results showed that TLR2 protein level was significantly inhibited (*p* < 0.05) (Figure 3A). The expression of PI3K/AKT-related proteins were investigated (Figure 3B), and MyD88 protein levels showed no significant changes at different time points (*p* > 0.05) (Figure 3C). Moreover, LTA lost its capacity to activate PI3K/AKT pathway (Figures 3D,E). The expression of TNF-α and IL-6 mRNA showed no significant differences compared with controls (*p* > 0.05) (Figures 3F,G). All these confirmed that TLR2 is required for LTA-induced inflammatory response in mammary epithelial cells.

### The Changes of Cellular and Exosomal miRNAs in Mammary Epithelial Cells Treated With Lipoteichoic Acid

**Figure 4:**
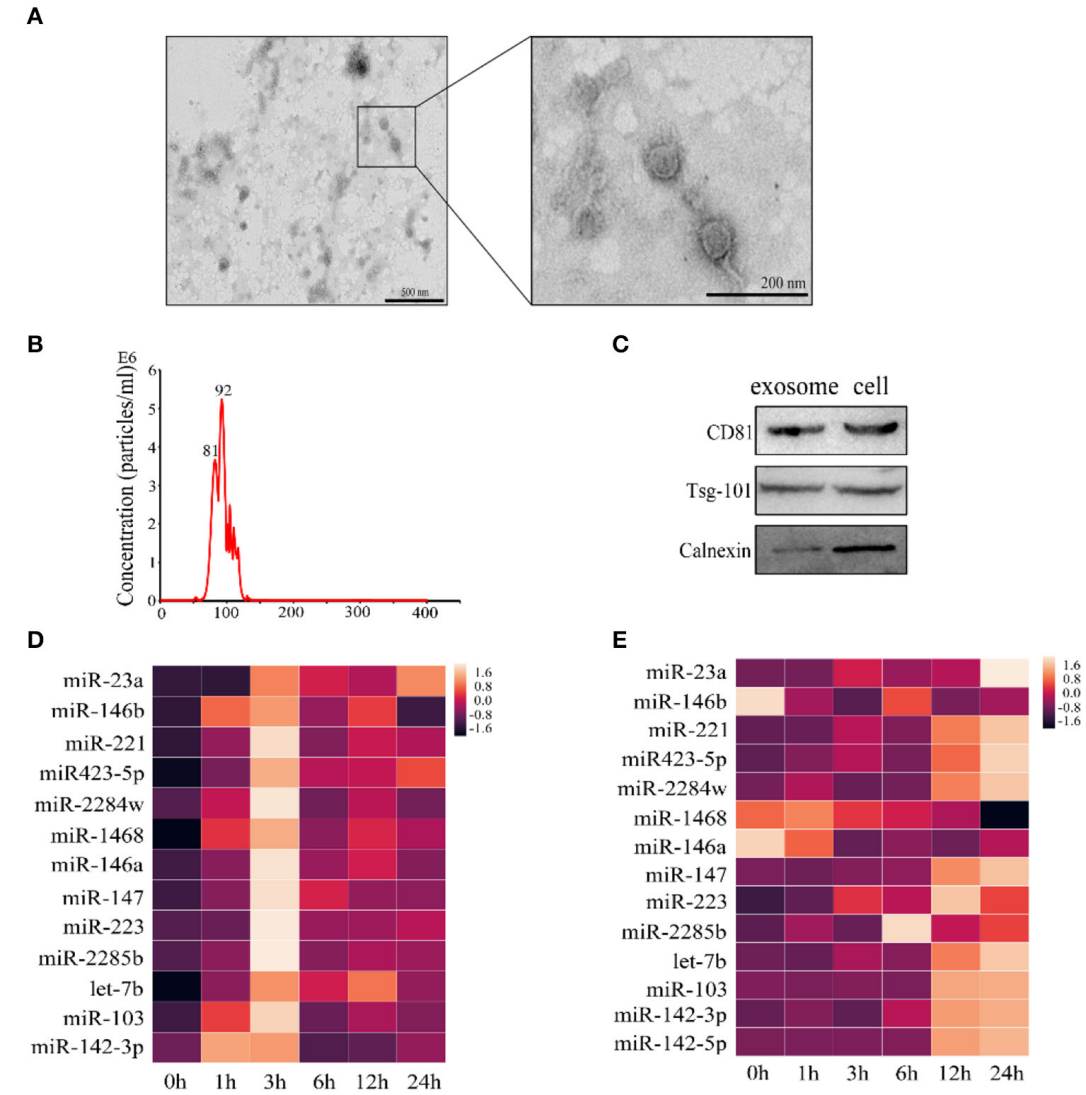
qRT-PCR of 14 miRNAs in Mac-T cells and exosomes. **(A)** TEM analysis of exosomes. **(B)** Nanosight depicting the size distribution of exosomes. **(C)** Western blot analysis of exosomal markers (*n* = 3). **(D)** The expression of miRNAs in Mac-T cells (*n* = 3). **(E)** The expression of miRNAs in Mac-T-derived exosomes (*n* = 3).

Results from recent studies showed that numerous miRNAs are involved in LTA-induced inflammatory response in mammary epithelial cells; however, few studies focused on exosomal miRNAs (29–32). We have screened 14 inflammation-related miRNAs in a previous study, including miR-23a, miR-146b, miR-221, miR-423-5p, miR-2284w, miR-1468, miR-146a, miR-147, miR-223, miR-2285b, let-7b, miR-103, miR-142-3p, and miR-142-5p. We further analyzed the changes of these miRNAs in LTA-infected Mac-T cells and cell-derived exosomes. Using TEM, we determined that Mac-T cell-derived exosomes presented a distinct membrane structure with about 100 nm diameter and physically homogeneous (Figure 4A). Our results demonstrated that the size of exosomes ranged from 40 to 150 nm by Nanosight (Figure 4B). Western blot showed that exosomes were positive for the expression of CD81 and Tsg-101 and negative for the expression of calnexin (Figure 4C). Then, qRT-PCR analysis indicated that the level of miRNAs increased and reached the highest at 3 h and then gradually decreased over time in Mac-T cells (Figure 4D). However, the expression pattern of the 14 miRNAs in exosomes was different from that of cells and changed non-regularly at different time points. Of all, the levels of 11 miRNAs were upregulated and three miRNAs were downregulated at 24 h (Figure 4E). In addition, miR-23a in cells and miR-221 in exosomes were expressed highest at 3 and 24 h, respectively. Thus, both cellular and exosomal miRNAs were affected in Mac-T cells treated with LTA at different times.

### miR-23a Regulates the Inflammatory Response in Mammary Epithelial Cells by Targeting Phosphoinositide 3-Kinase

**Figure 5:**
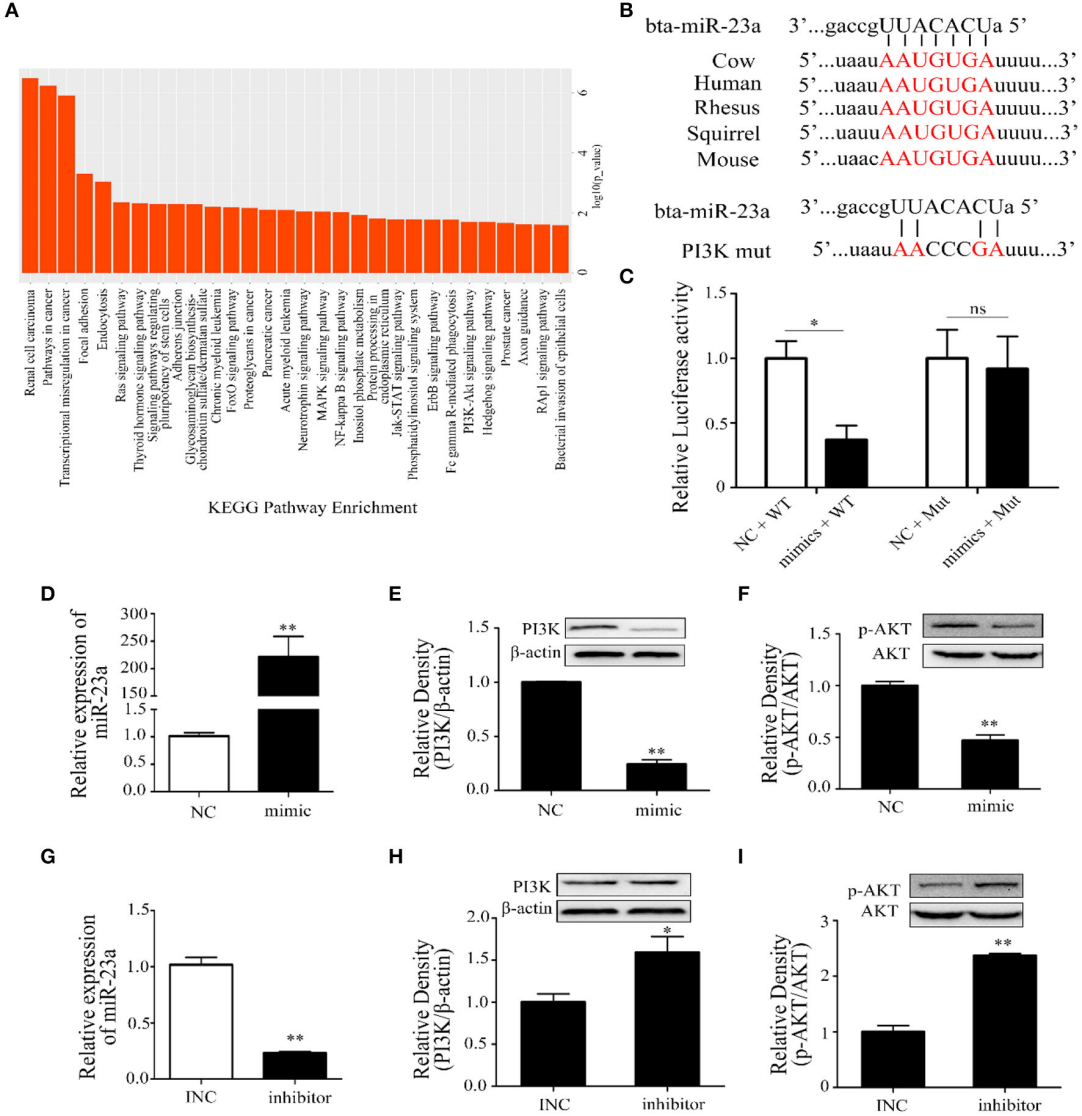
The regulation of miR-23a in lipoteichoic acid (LTA)-induced inflammatory responses. **(A)** Kyoto Encyclopedia of Genes and Genomes (KEGG) analysis of target genes of miR-23a. **(B)** The sequence information of miR-23a and target genes. **(C)** Dual-luciferase reporter analysis of miR-23a and phosphoinositide 3-kinase (PI3K) 3′-UTR (*n* = 3). **(D)** The expression of miR-23a in mimic-transfected cell (*n* = 3). **(E,F)** The expression of PI3K and AKT proteins after the transfection of miR-23a mimics (*n* = 3). **(G)** The expression of miR-23a in inhibitor-transfected cell (*n* = 3). **(H,I)** The expression of PI3K and AKT proteins after the transfection of miR-23a inhibitor (*n* = 3). *p < 0.05, **p < 0.01.

Thus, the target genes of miR-23a were screened and analyzed by using online databases and software. The KEGG results indicated that the target genes were mainly enriched in immune system and cancer pathways, such as renal cell carcinoma, pathways in cancer, transcriptional misregulation in cancer, FOXO, PI3K-AKT, MAPK, and NF-κB signaling pathways (Figure 5A). Through bioinformatic analysis, PI3K was considered as a potential target gene of miR-23a (Figure 5B). We further identified that miR-23a could directly bind to the 3′UTR of PI3K according to the result of dual-luciferase reporter assay (Figure 5C). miR-23a mimics and inhibitors were synthesized and transfected into Mac-T cells with Lipofectamine 3000, and the expression of miR-23a was confirmed by qRT-PCR. The results indicated that miR-23a level was significantly increased and decreased, respectively (Figures 5D,G). In addition, Western blot was employed to detect PI3K and p-AKT protein levels, and results showed that miR-23a mimics inhibited the expression of PI3K and p-AKT proteins (Figures 5E,F). Inversely, miR-23a inhibitors promoted the activation of PI3K/AKT by targeting PI3K (Figures 5H,I). These results proposed that miR-23a inhibited inflammatory responses by targeting PI3K in LTA-treated mammary epithelial cells.

### Lipoteichoic Acid-Treated Mac-T Cells Derived Exosomes Promote M1 Polarization of Bovine Macrophages

**Figure 6:**
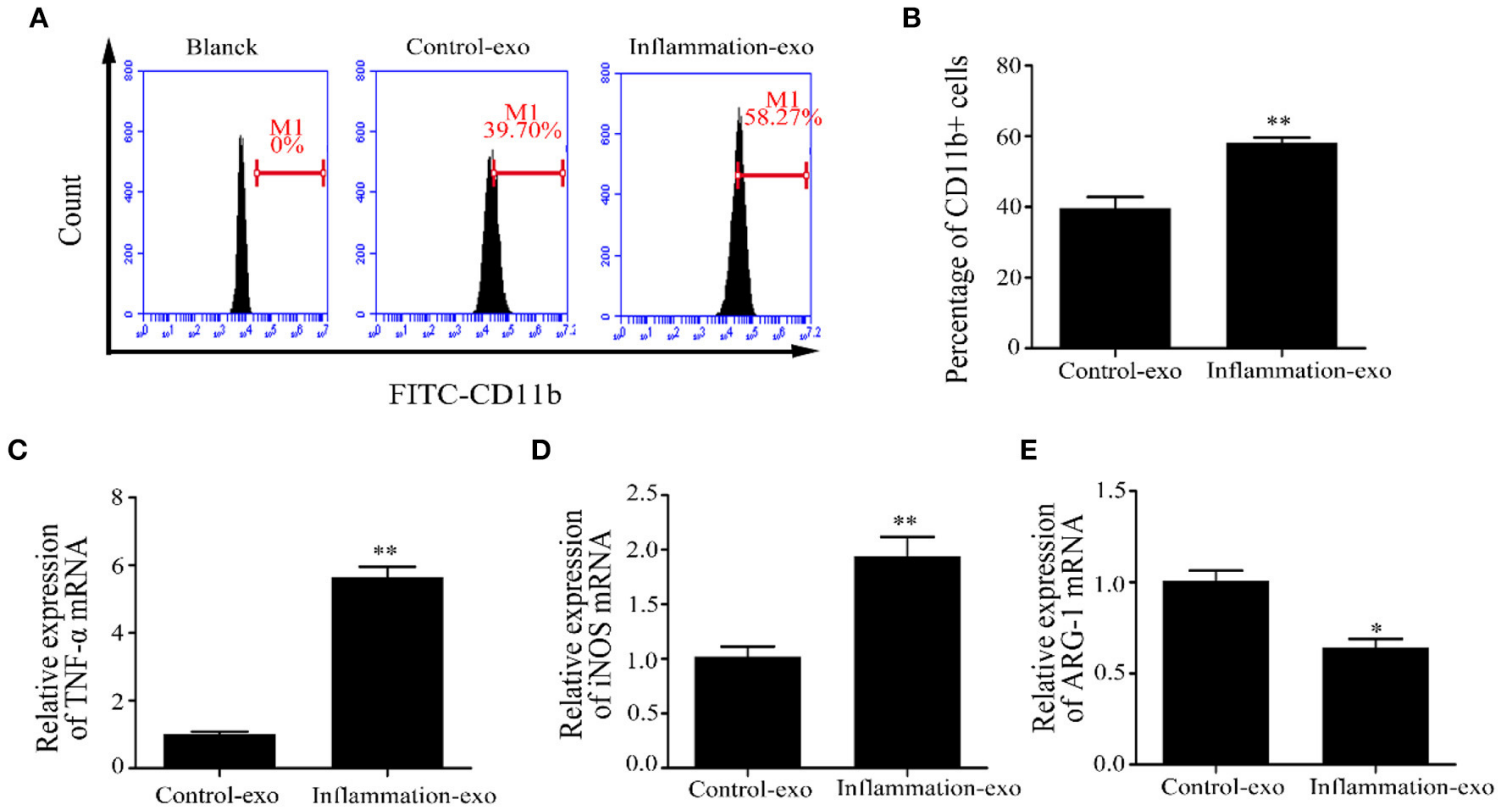
The polarization of bovine macrophages induced by exosomes (*n* = 3). **(A,B)** The expression of CD11b in macrophages after treatment with control-exo and inflammation-exo. **(C–E)** The expression of M1 and M2 macrophage markers, tumor necrosis factor (TNF)-α, inducible nitric oxide synthase (iNOS), and ARG-1, were detected by qRT-PCR. *p < 0.05, **p < 0.01.

To investigate the role of exosomal miRNAs, bovine macrophages were cultured and treated with exosomes derived from LTA-infected Mac-T cells at 0 (control-exo) and 24 h (inflammation-exo). The surface marker of M1 macrophage was detected by FCM, and the results showed that inflammation-exo could promote the expression of CD11b (Figures 6A,B). The expression of TNF-α and inducible nitric oxide synthase (iNOS) mRNA were significantly upregulated and Arg-1 mRNA level was downregulated compared with those in control-exo (Figures 6C–E). Therefore, we speculated that the exosomes, derived from LTA-treated Mac-T cells, played an important role in the polarization of M1 bovine macrophages.

## Discussion

In dairy cattle and goat, mastitis is an inflammation of mammary gland, which is commonly caused by pathogens. *S. aureus* is the most important pathogenic bacterium, frequently causing both clinical and subclinical mastitis (33). *S. aureus*-derived LTA was identified as a pathogen to stimulate an inflammatory response in several infectious diseases. Previous study showed that staphylococcal LTA activated the transcriptional responses to infections at various epithelial sites (34). Thus, the result further identified the important role of epithelial cells in the activation of inflammation (35). In addition, numerous studies analyzed the effects of *S. aureus* or LTA on the transcriptome miRNA profiling of mammary tissue or epithelial cells, but few studies further verified the functional regulatory mechanism of miRNAs. In the current study, we systematically investigated the activation of inflammatory responses in mammary epithelial cells treated with LTA at different time points and revealed a novel molecular mechanism through which exosomes induce the M1 polarization of macrophages. We clearly demonstrated that LTA was recognized by TLR2 and induced inflammatory responses via activating PI3K/AKT signaling pathway in mammary epithelial cells. And after treating Mac-T cells with LTA, miR-23a expression increased significantly and regulated inflammatory responses by targeting PI3K. After continuous infection for 12 h, cell-derived exosomes induced M1 polarization of macrophage by enhancing CD11b protein, TNF-α, and iNOS mRNA levels, along with the decrease of Arg-1 mRNA level.

In dairy cattle, the regulation of bacteria-derived components, LTA and lipopolysaccharide (LPS), in the activation of immune responses has been investigated. Previous studies demonstrated that LTA induces an inflammatory response via a TLR2-mediated signaling pathway known as MyD88-dependent. Finally, LTA could lead to the initiation of NF-κB and secretion of various cytokines, such as TNF-α, IL-6, IL-1, and IL-8 (36, 37). In this study, Mac-T cells were treated with LTA at different time points, and the secretion and expression of TNF-α and IL-6 were significantly increased at 6 h; these results indicate that the inflammatory responses were activated. The mRNA and protein levels of TLR2 and MyD88 were upregulated at 6 h with the initiation of PI3K/AKT pathway. However, TLR2, MyD88, PI3K, and p-AKT protein levels were downregulated at 24 h compared with 6 h. Similarly, the levels of MyD88, IκB-α, and NF-κB p65 crested at 12 h after LTA infection, followed by a continuous decline with an extended treated time of 24 h in RAW264.7 macrophages (38). Altogether, these findings illuminated that a long-term infection with LTA might gradually weaken the stimulation of TLR2-MyD88-mediated signaling pathway. In addition, TLR2 inhibitor, OxPAPC, effectively inhibited the activation of PI3K/AKT pathway, indicating that TLR2 is essential for LTA to induce inflammatory responses in mammary epithelial cells.

Recent studies showed that numerous miRNAs are differentially expressed after infection, both *in vivo* or *in vitro*, and play an important role in immunity and development (39, 40). Our previous study screened 14 inflammation-related miRNAs in bovine milk-derived exosomes infected with *S. aureus*. Therefore, we explored the expression patterns of these miRNAs in this study to further reveal the regulatory mechanism of miRNAs in mammary epithelial cells stimulated by LTA. Consistent with a previous study, miR-23a level was found significantly increased at 3 h after infection with LTA (39). And miR-23a inhibited an inflammatory response by targeting PI3K. However, miR-23a was found to be decreased in arthritis chondrocytes during rheumatoid arthritis pathogenesis and psoriatic arthritis, and miR-23a expression directly targeted inhibitor of kappa B kinase α (IKKα) to inhibit IL-17-mediated pro-inflammatory mediator expression (41, 42). These results indicated that instead of different miR-23a expression trends, it showed similar anti-inflammatory effects.

Exosomes have been identified to play important intercellular communications via transporting the cargoes, including proteins, mRNAs, and miRNAs, to recipient cells, in which the exosome-derived mRNA and miRNA could regulate gene expression (43). In the present study, the levels of 14 mature miRNAs were tested in LTA-treated Mac-T cells and exosomes at different time points. Our results indicated that the expression of the 14 exosomal miRNAs differentially upregulated or downregulated at 24 h, which further indicated that a long-time treatment with LTA could influence the sorting of miRNAs in mammary epithelial cell-derived exosomes. Similarly, exosomes were enriched in miRNAs that could target pro-inflammatory mRNAs, which were involved in key pathways, such as Janus kinase (JAK)/signal transducer and activator of transcription (STAT) signaling and NF-κB signaling in U937/Nef-EYFP cells (44). Additionally, we hypothesize that mammary epithelial cell-derived exosomes, treated with LTA for 24 h, transferred miRNAs to the target immunity-related cells and played critical roles in inflammation. Therefore, the different exosomes, control-exo and inflammation-exo, were cultured with bovine macrophages, respectively, and we found that inflammation-exo promoted the polarization of macrophage toward M1. Based on these results, we demonstrated that Mac-T cell miRNAs, as pro-inflammatory regulators, could be differentially sorted into exosomes and transferred into macrophages, which played important regulation of macrophage polarization.

In conclusion, our data suggested that 40 μg/ml LTA could activate TLR2/MyD88-mediated PI3K/AKT signaling pathway. Meanwhile, the expression pattern of cellular and exosomal miRNAs showed significant differences at different time intervals. Specially, LTA induced the highest increase of cellular miR-23a level, which inhibited inflammatory responses by targeting PI3K. Additionally, cell-derived exosomes played an important role in the M1 polarization of bovine macrophages.

## Data Availability Statement

The original contributions presented in the study are included in the article/supplementary material, further inquiries can be directed to the corresponding author.

## Author Contributions

MC and WF proposed the research thinking and designed the study. MC, XL, LD, DL, and YD contributed to the experiments and collection of data. HS, YL, and XL contributed to data analysis and provided the charts of this study. MC and XL drafted the manuscript. HS, LD, DL, YD, and YL contributed important opinions and suggestions on the manuscript. All the authors read and approved the final manuscript.

## Conflict of Interest

The authors declare that the research was conducted in the absence of any commercial or financial relationships that could be construed as a potential conflict of interest.
